# *Shigella* *flexneri* Regulator SlyA Controls Bacterial Acid Resistance by Directly Activating the Glutamate Decarboxylation System

**DOI:** 10.3389/fmicb.2018.02071

**Published:** 2018-08-31

**Authors:** Buyu Zhang, Longhao Ran, Mei Wu, Zezhou Li, Jiezhang Jiang, Zhen Wang, Sen Cheng, Jiaqi Fu, Xiaoyun Liu

**Affiliations:** Institute of Analytical Chemistry and Synthetic and Functional Biomolecules Center, College of Chemistry and Molecular Engineering, Peking University, Beijing, China

**Keywords:** *Shigella flexneri*, SlyA, acid resistance, glutamate decarboxylation, GadA

## Abstract

*Shigella flexneri* is an important foodborne bacterial pathogen with infectious dose as low as 10–100 cells. SlyA, a transcriptional regulator of the MarR family, has been shown to regulate virulence in a closely related bacterial pathogen, *Salmonella* Typhimurium. However, the regulatory role of SlyA in *S. flexneri* is less understood. Here we applied unbiased proteomic profiling to define the SlyA regulon in *S. flexneri*. We found that the genetic ablation of *slyA* led to the alteration of 18 bacterial proteins among over 1400 identifications. Intriguingly, most down-regulated proteins (whose expression is SlyA-dependent) were associated with bacterial acid resistance such as the glutamate decarboxylation system. We further demonstrated that SlyA directly regulates the expression of GadA, a glutamate decarboxylase, by binding to the promotor region of its coding gene. Importantly, overexpression of GadA was able to rescue the survival defect of the Δ*slyA* mutant under acid stress. Therefore, our study highlights a major role of SlyA in controlling *S. flexneri* acid resistance and provides a molecular mechanism underlying such regulation as well.

## Introduction

The bacterium *Shigella flexneri* (*S. flexneri*) is an intracellular pathogen that causes gut infection resulting in watery diarrhea or bacillary dysentery (Kotloff et al., 1999). It is estimated that there are over 160 million cases of shigellosis per year worldwide, leading to more than half a million deaths mostly associated with infants in developing countries (Kotloff et al., 1999). A major virulence determinant of *S. flexneri* is a large 230-kb plasmid that encodes the bacterial type III secretion system (T3SS) (Mattock and Blocker, 2017). Working as a molecular syringe, the T3SS is able to translocate an extensive repertoire of effector proteins into host cells, promoting initial bacterial internalization as well as intracellular replication. As a foodborne pathogen, *S. flexneri* infection of the gastrointestinal (GI) tract requires successful passage and survival through the highly acidic stomach. A rather striking feature of this bacterium is its extremely low infectious dose of as few as 10–100 bacterial cells. Therefore, bacterial acid resistance, in addition to its T3SS, constitutes another critical virulence determinant in *Shigella* spp.

Bacteria such as *S. flexneri* have evolved a plethora of strategies to cope with acid stress. A classical anti-acid mechanism of great importance is the amino acid (e.g., glutamate) decarboxylase-dependent systems (Jennison and Verma, 2007). In principle, protons are consumed by glutamate decarboxylation together with the release of CO_2_, thereby raising the cytoplasmic pH. GadA and GadB are the central enzymes in this process that catalyze the decarboxylating reaction (Bearson et al., 1997). Over the years, this important anti-acid mechanism has been well characterized (Hersh et al., 1996; De Biase et al., 1999; Castanie-Cornet and Foster, 2001; Foster, 2004); however, its transcriptional regulation still remains largely unclear in *S. flexneri*.

The MarR/SlyA family of transcription factors regulates a variety of bacterial cellular processes (George and Levy, 1983; Roper et al., 1993; Thomson et al., 1997; Srikumar et al., 1998; Galan et al., 2003). In *Salmonella* Typhimurium, for instance, SlyA has been shown to be an important regulator of bacterial virulence. The *slyA* deletion mutant exhibits a survival defect within macrophages as well as in a mouse model of infection (Libby et al., 1994; Buchmeier et al., 1997). Further transcriptome studies of the *slyA* deletion mutant in comparison to the wild-type strain revealed that it controls the expression of several known virulence genes (Navarre et al., 2005). In addition, many of the SlyA-controlled genes are also regulated by the PhoP-PhoQ two-component system (Navarre et al., 2005). In contrast, the regulatory role of SlyA in *S. flexneri* is less characterized thus far. In a pioneering study, Weatherspoon-Griffin and Wing (2016) recently reported the association of overexpressed SlyA with *S. flexneri* virulence. They also uncovered a survival defect of the *slyA* deletion mutant under acid stress, though the underlying mechanisms remain unclear (Weatherspoon-Griffin and Wing, 2016).

Here we performed unbiased proteomic profiling of a *S. flexneri* mutant lacking *slyA* in comparison to its parental strain. We found differential regulation of 18 proteins among over 1400 detected bacterial proteins. Notably, our data revealed SlyA-dependent expression of GadA and GadB (the glutamate decarboxylase), suggesting a direct link of this transcription factor to *S. flexneri* acid resistance. We provided further evidence that SlyA regulates the expression of Gad proteins by directly binding to the gene promoter regions. Importantly, *S. flexneri* mutants lacking *slyA* exhibited a severe survival defect under acid challenge and overproduction of the glutamate decarboxylase was able to restore, to a large extent, the bacterial anti-acid capacity of the *slyA* deletion mutant. Therefore, our study provides the first paradigm in bacteria that SlyA controls acid resistance by directly activating the glutamate decarboxylation system.

## Materials and Methods

### Bacterial Strains and Construction of Mutants

The *S.* *flexneri* serotype 2a 2457T (generously provided by Dr. Feng Shao from National Institute of Biological Sciences, Beijing, China) was used in this study, and the bacteria were routinely grown on trypticase soy agar plates with 1.5% agar and 30 μg/mL streptomycin at 37°C. A single colony picked from the plates was inoculated into trypticase soy broth (TSB) with 30 μg/mL streptomycin, and then the overnight culture was diluted 1:20 into 3 mL of TSB. The bacteria were harvested at indicated OD_600_ for different assays at 4°C. The *slyA* deletion mutant (Δ*slyA*) was constructed using the homologous recombination method as previously described (Liu et al., 2015). Successful deletion of a target gene was confirmed by both PCR analyses and sequencing. To construct the complementation strains harboring a plasmid-borne *slyA* or *gadA* gene in the Δ*slyA* background (Δ*slyA*+pSlyA or Δ*slyA*+pGadA), the *slyA* or *gadA* fragment was amplified, digested, and inserted into the pME6032 plasmid with an isopropyl β-D-1-thiogalactopyranoside (IPTG)-inducible promoter and a C-terminal 3 × FLAG tag. For β-galactosidase assays, the upstream region (1000 bp) of the *gadA* gene containing the promoter region was cloned and inserted into the pNN387 plasmid which contains a promoterless *lacZ* (Elledge and Davis, 1989). All primers and strains used in this study are listed in the **Supplementary Table S1**.

### Sample Preparation, Protein Digestion, and Stable Isotope Dimethyl Labeling

To uncover potential SlyA-regulated proteins, we performed proteomic analyses of the Δ*slyA* strain together with the wild-type (WT) strain. In brief, bacterial proteins were digested and isotopically labeled prior to two-dimensional LC-MS/MS analyses. Bacterial subculture was grown to an OD_600_ of ∼0.9 and pelleted at 3000 ×*g* for 5 min. Bacterial pellets were washed with ice-cold PBS (pH = 7.5) and resuspended in 1 mL of 8 M urea with 50 mM NH_4_HCO_3_. Then bacterial cells were lysed by sonication and protein concentration was measured by using a BCA protein assay kit (Solarbio, China). After diluted to one fourth with 50 mM NH_4_HCO_3_, 1 mg of proteins from each sample was digested with trypsin (Promega, United States) overnight at an enzyme-to-protein ratio of 1:50 (w/w) at 37°C. Resulting tryptic peptides were desalted and labeled on C18 Sep-Pak cartridges (Welch, China). For desalting, the columns were activated with 1 mL of acetonitrile (ACN) containing 0.1% (vol/vol) formic acid (FA) and equilibrated with 1 mL of 0.1% FA. Then the resulting tryptic peptides were loaded onto the columns. After washing with 1 mL of 0.1% FA, the on-column isotopic labeling experiments were performed as described previously (Boersema et al., 2009). Peptides on the column were repeatedly treated for three times by 1 mL of the labeling solution containing 100 mM triethyl ammonium bicarbonate (TEAB), 30 mM sodium cyanoborohydride (NaBH_3_CN), 0.2% formaldehyde (CH_2_O) or deuterated formaldehyde (CD_2_O). Peptides from WT and Δ*slyA* strains were labeled with CH_2_O and CD_2_O, respectively. After labeling, the peptides were washed once with 0.1% FA and eluted with 70% ACN plus 0.1% FA. Then light- and heavy-labeled peptides were pooled and vacuum dried for further offline fractionation.

### Offline High pH Reversed-Phase Fractionation and Nanoflow LC-MS/MS Analyses

Peptide samples were further fractionated using C18 Sep-Pak cartridges (Welch, China) under high pH conditions (10 mM NH_4_HCO_3_, pH 10). For peptide elution, the following percentages of ACN were used: 6, 9, 12, 15, 18, 21, 25, 30, and 35% (Lai et al., 2016). In total, nine fractions were collected and pooled into six samples (6+25%, 9+30%, 12+35%, 15%, 18%, and 21%). Samples were vacuum dried for subsequent LC-MS analyses. Nanoflow reversed-phase LC separation was carried out on an EASY-nLC 1200 system (Thermo Fisher Scientific). The capillary column (75 μm × 150 mm) with a laser-pulled electrospray tip (Model P-2000, Sutter Instruments) was home-packed with 4 μm, 100 Å Magic C18AQ silica-based particles (Michrom BioResources Inc., Auburn, CA, United States). The mobile phase was composed of Solvent A (97% H_2_O, 3% ACN, and 0.1% FA) and Solvent B (20% H_2_O, 80% ACN, and 0.1% FA). The LC separation was carried out at room temperature with the following gradient: Solvent B was started at 7% for 3 min, and then raised to 40% over 120 min; subsequently, Solvent B was rapidly increased to 90% in 2 min and maintained for 10 min before 100% Solvent A was used for column equilibration. Eluted peptides from the capillary column were electrosprayed directly into a hybrid linear ion trap-Orbitrap mass spectrometer (LTQ Orbitrap Velos, Thermo Fisher Scientific) for MS and MS/MS analyses in a data-dependent acquisition mode. One full MS scan (*m*/*z* 350–1200) was acquired and then MS/MS analyses were performed on the 10 most intense ions. The selected ions were fragmented by collision-induced dissociation (CID) in the ion trap with the following parameters: ≥+2 precursor ion charge, 2 Da precursor ion isolation window, and 35% normalized collision energy. Dynamic exclusion was set with repeat duration of 24 s and exclusion duration of 12 s.

### Proteomic Data Processing

Raw MS files were processed by MaxQuant (version 1.5.4.1) and searched against the *S.* *flexneri* (strain 301/serotype 2a) protein database (4103 sequences, downloaded from UniProt). The precursor mass tolerance was set at 20 ppm and the fragment mass tolerance was set at 0.8 Da. Maximum missed cleavage was set to 2. Dimethyl (K, N-term) and dimethyl (D_4_K, D_4_N-term) were set as variable modifications for light (L)- and heavy (H)-labeled samples, respectively. Oxidation (M) was set as a variable modification. Both peptide and protein assignments were filtered to achieve a false discovery rate (FDR) <1%. Only proteins with at least two unique plus razor peptides were quantified. The protein group lists were further processed by using the Perseus software (version 1.5.4.1) for the calculation of logarithmic values (log_2_) of the H- and L-labeled protein intensity ratio and the *p*-values. Proteins with ratios (H/L) >2.0 or <0.5 and *p*-values <0.05 were considered as significant difference between the WT and Δ*slyA* strains.

### Quantitative Real-Time PCR

Both the WT and Δ*slyA* strains were grown to an OD_600_ of ∼0.9 as described above. Total bacterial RNA from 1 mL of culture was extracted by using an EasyPure RNA Kit (TransGen Biotech, China) and then treated with DNase I. Reverse transcription of RNA was performed with TransScript One-Step gDNA Removal and cDNA Synthesis SuperMix (TransGen Biotech, China). RT-PCR analyses were carried out on an Applied Biosystems ViiA^TM^ 7 Real-Time PCR System by using UltraSYBR Mixture (Low ROX) (CWBIO, China). To quantitatively compare the levels of *gadA, hdeB, slp*, and *SF2991* transcripts, the housekeeping 16S rRNA gene was used for normalization. The mRNA levels were determined using the comparative threshold cycle number (2^-ΔΔC_t_^) method (Livak and Schmitge, 2001).

### β-Galactosidase Assays

*Shigella* cells were grown in TSB at 37°C to an OD_600_ of ∼1.0. Bacterial cells from 1.2 mL of culture were pelleted at 14,000 ×*g* for 2 min and then resuspended in 1.2 mL of Z buffer (60 mM Na_2_HPO_4_, 40 mM NaH_2_PO_4_, 10 mM KCl, and 1 mM MgSO_4_) plus 50 mM β-mercaptoethanol (freshly added). Subsequently, 30 μL of chloroform and 15 μL of 0.1% SDS were added and mixed upon vortexing. The assays were started by the addition of 240 μL of 4 mg/mL o-nitrophenyl-β-D-galactopyranoside (ONPG). Upon the observation of a faint yellow color, the reaction was quenched by the addition of 600 μL of 1 M Na_2_CO_3_ and the reaction time was recorded. Finally, samples were centrifuged at 14,000 ×*g* for 2 min, and the OD_420_ of the supernatant was recorded. Assay units were calculated as 1,000 × OD_420_/(OD_600_) (total reaction time).

### Expression and Purification of Recombinant Proteins

His_6_-tagged SlyA was expressed in the *E. coli* strain BL21 (DE3) harboring the appropriate plasmid. Briefly, 15 mL of the overnight culture was added in 300 mL LB broth and grown until the OD_600_ reached 0.6–0.8. Upon the addition of IPTG to a final concentration of 0.2 mM, bacterial culture was incubated further in a shaker at 18°C for 16–18 h. Bacterial cells (from 300 mL of culture) were lysed in 30 mL of ice cold PBS buffer via sonication and cell lysates were clarified by centrifugation at 5,000 ×*g* for 10 min three times at 4°C. His-tagged proteins were captured with Ni-NTA Resin (GenScript) and washed serially with PBS and 20 mM imidazole. Finally, proteins were eluted with 300 mM imidazole. Purified proteins were further dialyzed overnight in 25 mM Tris-HCl (pH 7.5), 150 mM NaCl, 5% (vol/vol) glycerol, and 1 mM DTT with at least two buffer changes.

### Electrophoretic Mobility Shift Assays (EMSAs)

The putative promoter sequence of *gadA*, a mutated version in which the SlyA-binding sequence (TTATCATGTTAA) was deleted and a DNA fragment of its coding sequence were amplified by PCR, purified with a Gel Extraction Kit (TransGen Biotech, China) and dissolved in water. Then purified SlyA proteins were incubated with 40 nM DNA fragments in 20 μL of binding buffer (10 mM Tris-HCl (pH 7.5), 100 mM KCl, 1 mM EDTA, 0.1 mM DTT, 5% v/v glycerol, and 10 μg/mL BSA). Molar ratios between DNA fragment and SlyA were set at 1:0, 1:30, 1:35, 1:40, 1:45, and 1:50 (indicated as 0, 1.2, 1.4, 1.6, 1.8, and 2.0 μM SlyA, respectively, in **Figure 3**). The reaction mixtures were incubated at room temperature for 30 min and then loaded onto 8% native polyacrylamide gels. Electrophoresis was performed using 0.5 × TBE buffer (44.5 mM Tris, 44.5 mM boric acid, and 1 mM EDTA) in ice bath. The gel was stained with Gel Stain (Yeasen, China) and photographed by using a Tanon-1600 Gel Image System (Tanon, China).

### Bacterial Acid Survival Assays

The overnight cultures of individual *Shigella* strains were diluted 1:20 into 3 mL of LB. When OD_600_ reached about 0.6, IPTG was added to a final concentration of 0.2 mM to induce the expression of plasmid-borne SlyA or GadA for 4 h. Then bacterial cells were diluted 1:50 in the acidified LB media (pH 2.5 adjusted with HCl). Viable cell counts were determined at 0, 1, and 2 h post acid challenge by serial dilution and plating on LB agar plates. At least three repetitions were performed for each experiment.

## Results

### Comparative Proteomic Profiling of *S. flexneri* Wild-Type and Its Isogenic Δ*slyA* Strains

**FIGURE 1:**
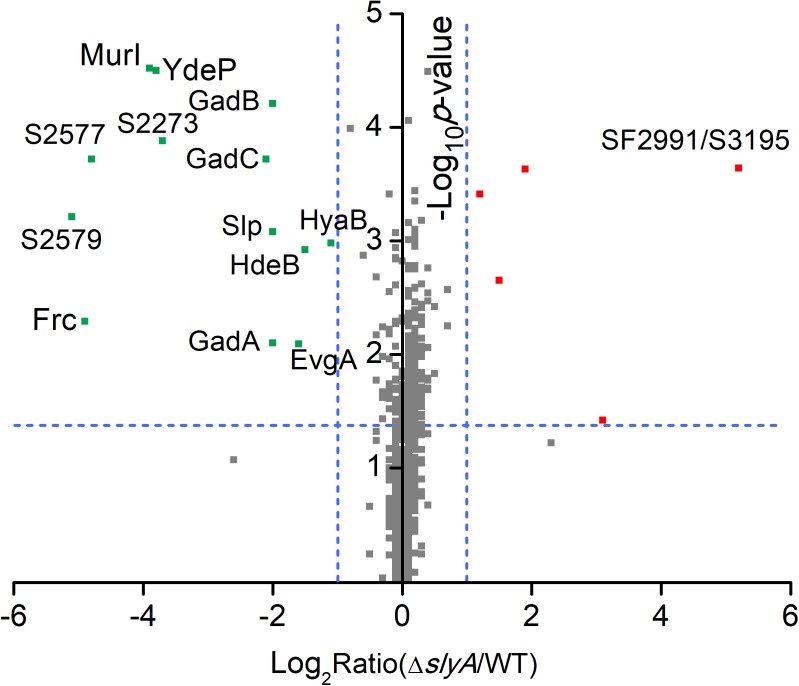
Quantitative proteomic data of *Shigella* WT and Δ*slyA* strains. A protein volcano plot was shown with the logarithmic values of the abundance ratios reported on the *x*-axis. The *y*-axis plots negative logarithmic *p*-values determined from the *t*-test on data from three biological replicates. Dotted lines denote two-fold (vertical) and *p* < 0.05 cutoff (horizontal). Proteins with homologous sequences may not be distinguished in LC-MS/MS experiments unless unique peptides were detected. As a result, the detection of SF2991 and S3195 is considered as a single protein identification (denoted as SF2991/S3195).

To study the SlyA regulon in *S. flexneri*, we carried out quantitative proteomic profiling of a bacterial strain lacking *slyA* (Δ*slyA*) and its parental wild-type (WT) strain. In total, we identified 1410 *S. flexneri* proteins from three biological replicates (i.e., six bacterial samples). A complete list of all identified bacterial proteins is provided in **Supplementary Table S2**. To get a global view of differentially expressed proteins, we plotted the ratio of protein abundance (Δ*slyA*/WT) as a function of calculated *p*-values (**Figure 1**). As seen in the protein-level volcano plot, the two proteomes are strikingly similar with vast majority of proteins lining up toward the center, indicating comparable expression levels of most proteins between two bacterial strains. By using the criteria described in the “Materials and Methods” section, 18 proteins were differentially regulated in the *slyA*-lacking strain, including 5 up-regulated and 13 down-regulated proteins (see **Table 1**). The most significant hit in the up-regulated proteins is SF2991/S3195, which is annotated as an outer membrane fluffing protein. The exact function of this protein has yet to be characterized. With respect to the down-regulated proteins, notably several proteins are well-known mediators of bacterial acid resistance such as GadA, GadB, GadC, and HdeB. The glutamate decarboxylation pathway represents arguably the most prominent example of bacterial anti-acid mechanisms. GadA and GadB are two glutamate decarboxylases catalyzing the proton-consuming reaction. MurI is a racemase catalyzing the interconversion of L-glutamate to D-glutamate, and therefore it is related to the GadAB-mediated bacterial anti-acid system.

**Table 1:** A list of altered *Shigella* proteins in the Δ*slyA* mutant.

	Gene	Protein description	Ratio	*p*-Value
Up-regulated	*SF2991/S3195*	Outer membrane fluffing protein	36.7	0.0002
	*S1803*	Putative membrane protein	8.5	0.0376
	*metE*	5-Methyltetrahydropteroyltriglutamate-homocysteine methyltransferase	3.7	0.0002
	*metF*	5,10-Methylenetetrahydrofolate reductase	2.8	0.0022
	*yaeC*	Lipoprotein	2.4	0.0004
Down-regulated	*S2579*	Uncharacterized protein	0.0	0.0006
	*frc*	Formyl-coenzyme A transferase	0.0	0.0051
	*S2577*	Putative enzyme	0.0	0.0002
	*murI*	Glutamate racemase	0.1	0.0000
	*ydeP*	Protein YdeP	0.1	0.0000
	*S2273*	Uncharacterized protein	0.1	0.0001
	*slp*	Outer membrane protein induced after carbon starvation	0.2	0.0002
	*gadB*	Glutamate decarboxylase beta	0.2	0.0001
	*gadA*	Glutamate decarboxylase alpha	0.2	0.0079
	*gadC*	Glutamate/gamma-aminobutyrate antiporter	0.3	0.0008
	*evgA*	Positive transcription regulator	0.3	0.0082
	*hdeB*	Acid stress chaperone HdeB	0.3	0.0012
	*hyaB*	Hydrogenase-1 large chain	0.5	0.0011

Furthermore, some of the other proteins that exhibited lower expression levels in the Δ*slyA* strain seem to be associated with bacterial acid resistance as well. For instance, YdeP was previously reported to confer acid resistance in *E. coli* (Masuda and Church, 2003). Interestingly, its regulator EvgA also appears in the list of those down-regulated proteins. In addition, the homologs of Frc and S2577 (i.e., YfdW and YfdU, respectively) were shown to play a role in *E. coli* acid resistance through a mechanism involving oxalate decarboxylation (Fontenot et al., 2013). Another protein, Slp, is an outer membrane protein that was depressed in the Δ*slyA* strain, consistent with a previous study of SlyA-regulated proteins in *E. coli* (Spory et al., 2002). Interestingly, we previously found that *S. flexneri* up-regulates Slp in response to acid stress (Yu et al., 2017). Taken together, these findings strongly suggest SlyA-dependent expression of many *Shigella* proteins associated with bacterial anti-acid mechanisms. Next we focused our attention on the characterization of the regulatory role of SlyA in mediating *S. flexneri* anti-acid capacities.

### Transcriptional Regulation of *S. flexneri* Anti-acid Genes by SlyA

**FIGURE 2:**
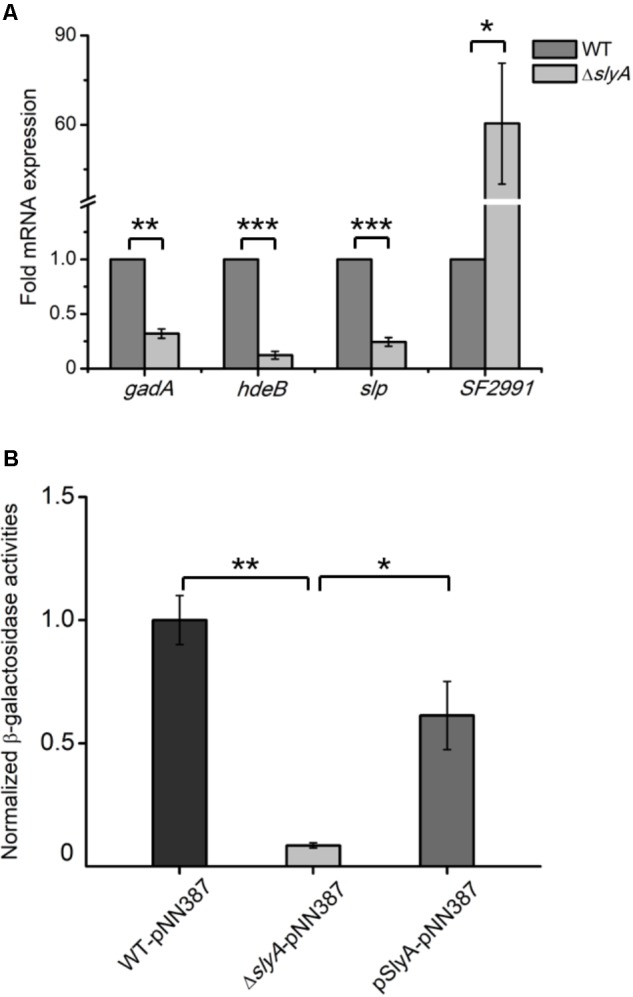
SlyA activates the expression of *gadA* at the transcriptional level. **(A)** qRT-PCR analyses of mRNA samples extracted from *Shigella* WT and Δ*slyA* strains. **(B)** Measurements of β-galactosidase activities in WT, Δ*slyA*, and Δ*slyA*+pSlyA strains harboring a plasmid encoding a promoterless *lacZ* fused with the *gadA* promoter. β-Galactosidase activities from three biological replicates are shown with values normalized to that of the WT strain. Asterisks indicate significant differences (^∗^*p* < 0.05, ^∗∗^*p* < 0.01, and ^∗∗∗^*p* < 0.001).

Proteomic observations of SlyA-dependent production of those anti-acid proteins prompted us to determine if SlyA, as a transcription factor, exerts its regulatory role on the transcript level. We carried out qRT-PCR measurements on some representative genes whose protein products were differentially expressed in the Δ*slyA* strain including *gadA, hdeB, slp*, and *SF2991* (**Figure 2A**). Consistent with the down-regulation of their corresponding proteins, the mRNA levels of *gadA, hdeB*, and *slp* were profoundly lower in the *slyA*-deletion mutant than those in its parental strain. Additionally, the transcript of *SF2991*, a SlyA-repressed gene, was found to be substantially higher (∼60-fold) in the mutant strain. Unlike *gadA* and *slp*, the fold difference of *hdeB* and *SF2991* transcript levels is markedly larger than that of their protein levels between the Δ*slyA* and WT strains, indicating potential mechanisms of post-transcriptional regulation.

Given the prominent role of the glutamate decarboxylation system in acid resistance, next we further explored the transcriptional control of *gadA* by SlyA by utilizing a β-galactosidase-based reporter assay. We constructed a *lacZ*-expressing plasmid in which its promoter region was replaced by that of *gadA* and transformed this plasmid into *Shigella* strains of different genetic backgrounds (WT, Δ*slyA*, and Δ*slyA*+pSlyA). By measuring the enzymatic activities of expressed β-galactosidase *in vitro*, we could assess the promoter activity of the *gadA* gene in different strains. As shown in **Figure 2B**, β-galactosidase activity decreased substantially (by at least an order of magnitude) in the Δ*slyA* mutant relative to that in its parental strain, indicating that the promoter activity of *gadA* is strongly dependent on SlyA. Furthermore, ectopic expression of SlyA in the Δ*slyA* mutant largely restored the promoter activity to the wild-type level. Taken together, these results established that SlyA positively regulates the expression of *gadA* on the transcript level and likely other *S. flexneri* genes involved in acid resistance as well.

### Direct Binding of *S. flexneri* SlyA to the Promoter Region of the *gadA* Gene

**FIGURE 3:**
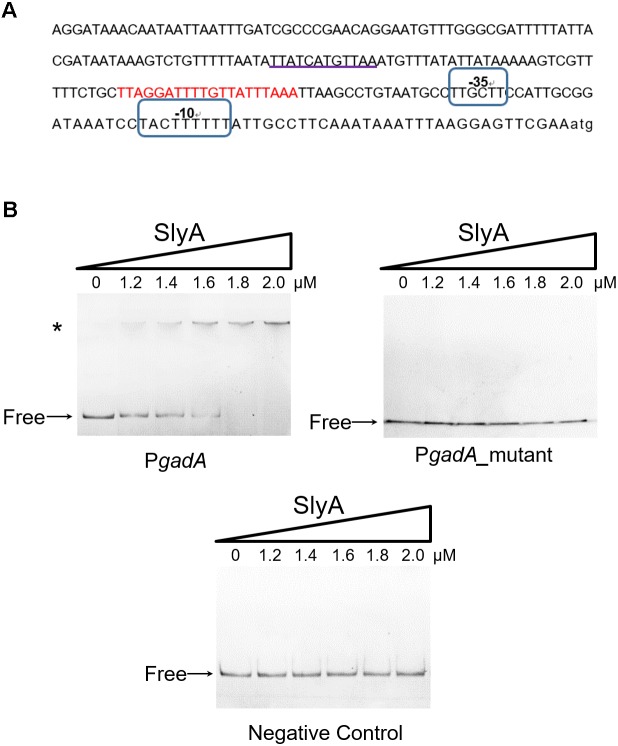
SlyA directly binds to the promoter region of the *gadA* gene. **(A)** Bioinformatics analysis of the *gadA* promoter region (P*gadA*). The sequence of the *gadA* promoter region of 224 bp upstream of the start codon is shown and the putative SlyA-binding site is underlined. Putative –10 and –35 elements are circled and the *gad* box is indicated in red. **(B)** EMSA experiments determined the direct binding of SlyA to P*gadA*. Different concentrations of purified SlyA (ranging from 0 to 2 μM) were incubated with P*gadA*, P*gadA* lacking the SlyA-binding site (P*gadA*_mutant) or negative control DNA prior to electrophoretic separation. “Free” indicates free DNA and the asterisk indicates DNA-protein complexes.

SlyA is a transcriptional regulator in the MarR family with a classical helix-turn-helix (HTH) DNA-binding domain (Ellison and Miller, 2006). Next we sought to determine if SlyA exerts a direct regulatory role in GadA expression by using electrophoretic mobility shift assays (EMSAs). Recombinantly purified SlyA proteins were incubated with various concentrations of DNA fragments spanning the *gadA* promoter region (the promoter region and putative SlyA-binding site are shown in **Figure 3A**). Electrophoretic experiments demonstrated that with increasing levels of SlyA a greater fraction of DNA fragments exhibited a mobility shift (**Figure 3B**). In contrast, when DNA fragments containing a partial sequence of *gadA*-coding region were used as a negative control, we did not observe any retardation in electrophoretic mobility at all concentrations of SlyA. Remarkably, deletion of the putative SlyA-binding site in the *gadA* promoter sequence completely abolished its binding to SlyA (**Figure 3B**). Together, these findings reveal that SlyA directly controls the expression of GadA by physically associating with the gene promoter region.

### *S. flexneri* SlyA Mediates Acid Resistance by Regulating the Expression of Gad Proteins

**FIGURE 4:**
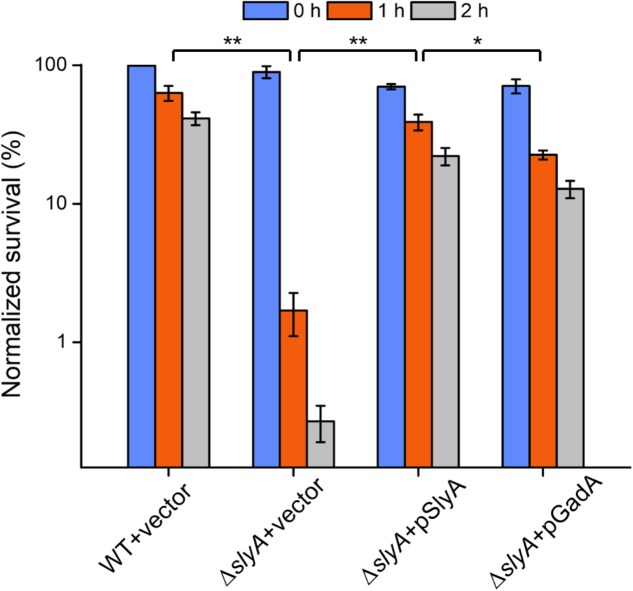
Acid survival assays of various *Shigella* strains. Survival percentages of *Shigella* wild-type and Δ*slyA* strains complemented with either empty vectors or SlyA/GadA-expressing plasmids were shown. Bacteria were incubated in acidic conditions for 0, 1, and 2 h and assayed for viability. Asterisks indicate significant differences (^∗^*p* < 0.05 and ^∗∗^*p* < 0.01).

Given its direct regulatory role in the glutamate decarboxylation system, we hypothesized that SlyA may modulate *Shigella* acid resistance. To test our hypothesis, we challenged the Δ*slyA* strain in highly acidic conditions (pH 2.5) for different durations (1 and 2 h) and then determined bacterial viability by CFU assays (**Figure 4**). Compared to its parental WT strain, upon 1 and 2 h of acid challenge the *slyA*-deletion mutant exhibited substantially lower survival percentages (>50-fold and 300-fold less, respectively). Furthermore, we were able to rescue such a survival defect by reintroducing a functional copy of *slyA* into the Δ*slyA* strain (Δ*slyA*+pSlyA). Next, we sought out to determine if the regulation of bacterial acid resistance by SlyA is largely accomplished by its control of the glutamate decarboxylation pathway (e.g., the expression of GadA). To explore this possibility, we further constructed a bacterial strain harboring a copy of plasmid-borne *gadA* in the Δ*slyA* background. Remarkably, ectopic expression of GadA can restore, to a large extent, bacterial acid resistance of the Δ*slyA* strain to the levels of the complementation strain (Δ*slyA*+pSlyA) as well as the WT bacteria. Nonetheless, we do note a minor difference in the rescuing ability of two complementation strains as suggested by the slightly lower (yet significant) survival percentage of the Δ*slyA* strain ectopically producing GadA than the SlyA-complemented mutant. Taken together, these data suggest *Shigella* SlyA plays a crucial role in bacterial acid resistance and importantly such regulation is mostly exerted through its control of the glutamate decarboxylation pathway.

## Discussion

The MarR/SlyA family of transcriptional regulators has been identified in a variety of Gram-negative bacteria. In *Salmonella* Typhimurium, SlyA was reported to contribute to bacterial virulence by exerting a regulatory effect on SsrB (Navarre et al., 2005), a master regulator of *Salmonella* pathogenicity island 2 (SPI-2). Consistent with this notion, our previous work showed concurrent induction of *S.* Typhimurium SlyA and SPI-2-encoded virulence proteins during infection of epithelial cells (Liu et al., 2015, 2017). In addition to virulence, SlyA has been associated with other aspects of *S.* Typhimurium physiology including resistance to antimicrobial peptides (Navarre et al., 2005) and oxidative stress (Buchmeier et al., 1997). Unlike in *S.* Typhimurium, the regulatory role of SlyA in *S. flexneri* has been less defined. Previously, Weatherspoon-Griffin and Wing (2016) carried out the initial characterization of *S. flexneri* SlyA and identified a role in bacterial virulence as well. Our current study aims to examine the SlyA regulon in *S. flexneri* by using an unbiased proteomic approach. Quantitative profiling of a *S. flexneri* mutant lacking *slyA* in comparison to its parental strain led to the discovery of SlyA-dependent expression of many proteins associated with acid resistance such as GadA/B, HdeB, and Slp. Interestingly, the implication of SlyA in bacterial anti-acid defense was reported in Wing’s study, though the exact mechanism was not clarified (Weatherspoon-Griffin and Wing, 2016).

Among many bacterial anti-acid strategies, the glutamate decarboxylase-dependent pathway is one of the most effective systems for acid resistance. This system consists of three proteins, GadA, GadB, and GadC, all of which were down-regulated in the *slyA* deletion mutant. We further verified SlyA-dependent transcription of *gadA* by both mRNA measurements and β-galactosidase-based reporter assays. Importantly, we found SlyA directly regulates the expression of *gadA* by binding to its promoter region. After establishing the expression of GadA (and likely the entire Gad system) in a SlyA-dependent manner, we further examined the anti-acid capacities of the *slyA*-deletion mutant. Consistent with the previous report, the strain lacking *slyA* exhibited a severe survival defect upon acid challenge. Complementation with a SlyA-expressing plasmid was able to restore its anti-acid capacities to the level of the wild-type bacteria. If the glutamate decarboxylase-dependent pathway constitutes the major anti-acid system under the control of SlyA, we reasoned that the introduction of a GadA-expressing plasmid would rescue, at least in part, the survival defect of the *slyA*-deletion mutant under acid stress. Indeed, we found overproduction of GadA in the *slyA*-deletion strain largely restored its anti-acid capacities, though at slightly lower levels than the Δ*slyA* strain harboring a SlyA-producing plasmid as well as the WT bacteria. Such observations are in fact consistent with our proteomic data suggesting SlyA-dependent activation of multiple anti-acid pathways in addition to the Gad system. In other words, SlyA may regulate *S. flexneri* acid resistance through downstream proteins other than GadA.

*Shigella flexneri* *gadB* and *gadC* genes are located in a transcriptional unit different from that of *gadA*. In *E. coli*, a number of regulators have been identified to control the transcription of these *gad* genes, such as GadE (Ma et al., 2003a), GadW (Tucker et al., 2003), GadX (Tucker et al., 2003), PhoP (Zwir et al., 2005), YdeO (Ma et al., 2004), EvgA (Masuda and Church, 2003; Ma et al., 2004), RpoS (De Biase et al., 1999), and Crp (Ma et al., 2003b). Notably, GadE plays a central role in transcriptional regulation and activates the expression of *gad* genes by directly binding to the *gad* box (shown in **Figure 3A**), a 20-bp sequence located 63-bp upstream of the transcriptional start sites of *gadA/B* and *gadC* (Castanie-Cornet and Foster, 2001). Most of the other transcription factors above were found to regulate the expression of Gad proteins via the control of GadE. For instance, two AraC-like regulators, GadW and GadX, whose genes are located downstream of *gadA*, directly activate the transcription of *gadE* and hence the expression of *gadA/B* and *gadC* (Ma et al., 2002; Tramonti et al., 2002, 2003). However, under certain conditions, GadW and GadX can function as repressors by competitively binding to the *gad* box sequences (Castanie-Cornet and Foster, 2001; Ma et al., 2002; Tramonti et al., 2002). Thus, our discovery that *S. flexneri* SlyA regulates the transcription of *gadA* by directly binding to its promoter further expands the repertoire of regulators controlling the glutamate decarboxylase-dependent system. Previously, *E. coli* SlyA was also reported to regulate the expression of GadA and other proteins involved in acid resistance such as HdeA and HdeB (Spory et al., 2002). It is therefore tempting to speculate that the regulation of acid resistance by SlyA may be a conserved mechanism shared by many bacterial species.

**FIGURE 5:**
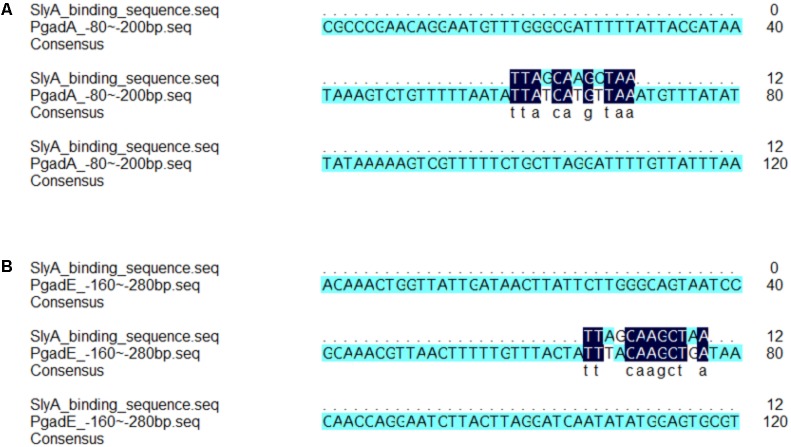
Alignment analyses revealed consensus SlyA-binding sequence in the promoter regions of the *gadA* and *gadE* genes. The alignment of consensus SlyA-binding sequence and fragments of the promoter of *gadA* **(A)** and *gadE* **(B)** was performed by the DNAMAN software.

**FIGURE 6:**
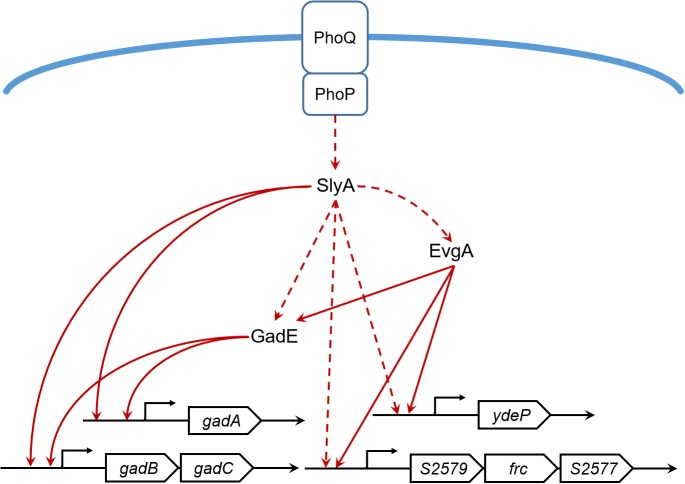
The SlyA regulatory network of *S. flexneri* acid resistance. Solid lines indicate direct or experimentally verified transcriptional regulation. Dashed lines indicate hypothetical regulation inferred from our proteomic results or literatures.

Our proteomic data also revealed other SlyA-regulated proteins involved in acid resistance such as EvgA, YdeP, Frc, and S2577. EvgA is a member of the two-component regulatory system EvgS/EvgA, which activates the transcription of other regulators such as YdeO and GadE, thereby leading to increased expression of the Gad system (Ma et al., 2004). YdeP is a putative oxidoreductase under the control of EvgA. Intriguingly, overexpression of this enzyme in *E. coli* enhanced bacterial acid resistance (Masuda and Church, 2003). Frc and S2577 are homologous to the formyl-CoA transferase YfdW and the oxalyl-CoA decarboxylase YfdU in *E. coli*, respectively. Previously Fontenot et al. (2013) reported the contribution of these two enzymes to bacterial anti-acid capacities involving oxalate decarboxylation. Interestingly, the *yfdXWUVE* operon is also controlled by EvgA (Masuda and Church, 2002), and *yfdX* encodes a protein homologous to S2579, another down-regulated protein in our dataset. Given that EvgA regulates Frc, S2577, and S2579 in addition to the Gad system, it would be tempting to determine whether SlyA exerts a regulatory role in EvgA and thus indirectly influences the expression of the *yfd* operon. Furthermore, by using the *gadA* promoter as a positive control, the alignment of the SlyA-binding consensus (Stapleton et al., 2002) and the *gadE* gene sequence suggests direct binding of SlyA to the *gadE* promoter region (**Figure 5**). Considering the complicated regulatory network, we propose that SlyA might be a global transcriptional regulator of *Shigella* acid resistance (**Figure 6**), though experimental validation of SlyA-exerted regulatory roles in other known mediators of acid resistance would be highly desired in the future. Furthermore, given the direct binding of PhoP to the SlyA promoter in *Salmonella* Typhimurium (Shi et al., 2004), it is conceivable that SlyA is activated by other transcriptional factors such as PhoP rather than direct sensing of the environmental pH. Indeed, *Shigella* *slyA* promoter was also reported to respond to Mg^2+^ and be positively regulated by PhoP (Weatherspoon-Griffin and Wing, 2016).

As a final note, we found insignificant difference in most T3SS-related proteins in the *slyA* deletion mutant (**Supplementary Table S3**), although Weatherspoon-Griffin and Wing (2016) suggested a potential role of SlyA in mediating *S. flexneri* virulence. In their previous work, ectopic expression of SlyA was found to increase the promoter activity of certain virulence genes and also rescue a virulence-associated phenotype. We reason that such observations may be resulted from the overproduction of SlyA from a high-copy-number plasmid, thereby differing to some extent from our proteomic measurements in which SlyA was expressed at physiological levels. Therefore, we propose that SlyA is likely to be a global transcriptional regulator of bacterial acid resistance in *S. flexneri*, which is in contrast to virulence regulation in *S.* Typhimurium.

## Data Availability

The proteomics data reported in this paper have been deposited to the iProX database (URL: http://www.iprox.org/page/HMV006.html) under the accession number IPX0001250001.

## Author Contributions

BZ and LR conducted most of the experiments with the assistance from MW, ZL, JJ, ZW, and SC. JF, BZ, LR, and XL wrote the manuscript with editorial inputs from all authors.

## Conflict of Interest Statement

The authors declare that the research was conducted in the absence of any commercial or financial relationships that could be construed as a potential conflict of interest.

## Supplementary Material

TABLE S1 All strains and primers used in this study.

TABLE S2 All identified bacterial proteins.

TABLE S3 Virulence proteins identified in the proteomic profiling.
